# Maintenance Liposomal Doxorubicin Following Induction Doxorubicin in Soft Tissue Sarcoma: A Single-Center Observational Study

**DOI:** 10.3390/curroncol33050260

**Published:** 2026-04-30

**Authors:** Alexandre Da Silva Faco, Mohammad Amin Salehi, Mohammad Hassan Hodroj, Kaveh Mozafari-Lorestani, Feras A. Moria, Jonathan Noujaim, Ramy R. Saleh

**Affiliations:** 1 McGill University Health Centre, Montreal, QC H4A 0B1, Canada; alexandre.faco@mail.mcgill.ca (A.D.S.F.J.); mohammad.salehi@rimuhc.ca (M.A.S.); mohammad.hodroj@mail.mcgill.ca (M.H.H.); kaveh.mozafari-lorestani@mail.mcgill.ca (K.M.-L.); 2 Faculty of Medicine, King Abdulaziz University, Jeddah 21589, Saudi Arabia; fmoria@kau.edu.sa; 3 Hôpital Maisonneuve-Rosemont, Montréal, QC H1T 2M4, Canada; jonathan.noujaim.med@ssss.gouv.qc.ca

**Keywords:** Doxorubicin, soft tissue sarcoma, liposomal doxorubicin, maintenance, systemic therapy

## Abstract

Maintenance chemotherapy prolongs progression-free survival (PFS) in several malignancies, but its role in soft tissue sarcoma (STS) remains unclear. Conventional doxorubicin is limited by cumulative toxicities. Liposomal doxorubicin may allow prolonged therapy. We evaluated outcomes of patients treated with maintenance liposomal doxorubicin following induction doxorubicin. This was a single-center retrospective study using the Quebec Sarcoma Registry from 2015 to 2025. Eligible patients had histologically confirmed STS and received doxorubicin-based induction therapy. Those achieving disease control were treated with maintenance liposomal doxorubicin. Clinical variables, adverse events, and outcomes were collected, and PFS and overall survival (OS) were estimated using the Kaplan–Meier method. Twenty-four patients were included (median age 61 years; 62.5% male), with leiomyosarcoma and undifferentiated pleomorphic sarcoma as the most frequent histologies. The median induction exposure was six cycles, and maintenance treatment was administered for a median of five cycles. Median PFS was 11.4 months, and median maintenance PFS was 6.1 months. Infusion reactions and hematologic toxicities were the most common adverse events. Median OS was 60.2 months. Maintenance liposomal doxorubicin was feasible, well-tolerated, and associated with encouraging disease control in selected patients, supporting further prospective evaluation.

## Simple Summary

Soft tissue sarcomas are rare cancers often treated with chemotherapy such as doxorubicin in the advanced or metastatic setting, but their use is limited by cumulative toxicity. It is unclear whether continuing treatment after initial disease control can improve outcomes. Liposomal doxorubicin is a modified formulation that may be better tolerated and allow longer treatment. In this study, we evaluated patients who received liposomal doxorubicin after responding to initial chemotherapy. We found that this approach was feasible, generally well tolerated, and associated with disease control in some patients. Side effects were manageable, and some patients were able to continue treatment for extended periods. These findings suggest that maintenance treatment with liposomal doxorubicin could be a promising strategy for selected patients with advanced disease. Further prospective studies are needed to confirm its benefit and to better define which patients are most likely to benefit.

## 1. Introduction

About 1% of adult malignancies are soft tissue sarcoma (STS), a rare and diverse group of mesenchymal malignancies [1]. Despite improvements in multimodal therapy, outcomes for patients with metastatic disease remain poor, with reported median survival of 12–18 months and five-year survival rates of 15–25% [2,3,4,5,6]. Doxorubicin remains the standard first-line therapy, either alone or in combination with other agents, such as ifosfamide [7,8]. However, treatment duration is limited, compromising long-term results by cumulative toxicities, especially cardiotoxicity and myelosuppression [9,10,11].

As seen in non-small-cell lung cancer and ovarian carcinoma, maintenance chemotherapy has become a viable strategy to extend disease control following an initial response [12,13]. Nevertheless, there is limited evidence supporting a survival advantage in STS, so the benefits of maintenance chemotherapy remain unknown. Moreover, patient tolerability and quality of life are threatened by the chronic toxicities linked to ongoing cytotoxic exposure [14].

Pegylated liposomal doxorubicin, an alternate formulation of conventional doxorubicin, has been proposed to lessen systemic toxicity while maintaining efficacy [15]. Encapsulation in liposomes alters the drug pharmacokinetics, enhances tumor-specific delivery, and minimizes cardiotoxicity [16]. When used as maintenance therapy, liposomal doxorubicin has demonstrated favorable safety and efficacy in other solid tumors, including ovarian and breast cancers [17,18,19]. These properties make it an attractive candidate for maintenance treatment in STS.

We conducted an observational study to describe the clinical outcomes, toxicities, and feasibility of liposomal doxorubicin as maintenance therapy following induction doxorubicin in patients with soft tissue sarcoma.

## 2. Materials and Methods

This was a single-center, retrospective observational study using data from the Quebec Sarcoma Registry (SaRC-Q), a centralized database that collects, manages, and analyzes data on sarcoma cases in Quebec [20]. The study was approved by the McGill University Health Centre (MUHC) Research Ethics Board. All data were de-identified, and all procedures adhered to the Tri-Council Policy Statement: Ethical Conduct for Research Involving Humans (2022) [21].

Eligible patients had histologically confirmed STS and received maintenance liposomal doxorubicin following doxorubicin-based induction chemotherapy. Between July 2015 and June 2025, 86 patients with STS were treated with liposomal doxorubicin at the MUHC; 24 patients were included in this study based on their exposure to maintenance therapy, while the remaining patients were excluded because liposomal doxorubicin was administered outside the maintenance setting (e.g., in later-line therapy or alternative treatment contexts).

Patients qualified for maintenance if they achieved disease control, defined as complete response, partial response, or stable disease, or were deemed to have derived clinical benefit from induction therapy based on the treating physician’s clinical judgment. Clinical benefit was pragmatically defined in the real-world setting and could include cases with radiologic progression in the presence of clinical stability, symptom control, or slow disease kinetics. At our institution, maintenance liposomal doxorubicin is routinely offered to all patients who achieve clinical benefit after induction therapy, in accordance with institutional practice. Patients who elect a treatment break do not proceed to maintenance therapy. Induction consisted of single-agent doxorubicin or combination regimens (e.g., with ifosfamide). Maintenance liposomal doxorubicin was typically administered at 40 mg/m^2^ every 28 days. Patients with incomplete data or non-soft tissue sarcomas were excluded.

Data extracted from the SaRC-Q database included demographics, tumor characteristics, treatment details, adverse events, and outcomes, including progression-free survival (PFS), maintenance progression-free survival (mPFS), overall survival (OS), and objective response rate (ORR). PFS was defined as the time from initiation of induction doxorubicin to progression or death. Maintenance progression-free survival (mPFS) was defined as the time from initiating liposomal doxorubicin to progression or death. OS was defined as the time from STS diagnosis to death from any cause. Tumor response and progression were assessed per RECIST v1.1 [22]. Adverse events were graded according to CTCAE v5.0 [23]. Cardiac function was monitored as part of the institutional standard of care, typically with multiple gated acquisition (MUGA) scans performed every 12–16 weeks during treatment. However, given the retrospective design, cardiac assessments were not standardized and were not consistently available for all patients; as such, cardiovascular data were not formally analyzed.

Descriptive statistics summarized baseline characteristics and treatment parameters. PFS and OS were estimated using the Kaplan–Meier method. Subgroup comparisons used log-rank tests and Cox proportional hazards models. Multivariable Cox regression models included age and ECOG performance status as covariates. Missing data were minimal and handled using complete-case analysis.

## 3. Results

### 3.1. Patient Characteristics

Twenty-four patients were included, with a median age of 61 years (range 36–82); 15 patients (62.5%) were male (Table 1). At diagnosis, 62.5% had localized disease (and later progressed) and 37.5% had metastatic disease. Performance status was generally preserved, with a median Eastern Cooperative Oncology Group (ECOG) of 1 (Figure S1) [24]. The most common histologic subtypes were leiomyosarcoma and undifferentiated pleomorphic sarcoma (UPS) (*n* = 7 each, 29.2%), followed by malignant peripheral nerve sheath tumor (MPNST) and solitary fibrous tumor (*n* = 2 each, 8.3%). The remaining histologies accounted for six cases (25.0%). Median follow-up from diagnosis was 23.5 months.

### 3.2. Treatment Exposure

All patients received doxorubicin-based induction therapy, with a median of six induction cycles (range 1–12). Overall median cumulative doxorubicin exposure was 435 mg/m^2^ (range 75–806 mg/m^2^). Six patients (25.0%) received combination regimens, most commonly doxorubicin plus ifosfamide, while the remainder received single-agent doxorubicin. The best response to induction therapy was partial response in nine patients (37.5%), stable disease in 13 (54.2%), and progressive disease in two (8.3%). Notably, the two patients with progressive disease were considered to have derived clinical benefit, which justified continued treatment and, at the treating physician’s discretion, they subsequently received maintenance therapy (Table 2)

Maintenance liposomal doxorubicin was administered at 40 mg/m^2^ every 28 days in all but one patient (96%) and was continued until disease progression, unacceptable toxicity, or physician or patient decision for a median of five cycles (Figure S2). Dose reductions were required in two patients (8.3%), and treatment interruptions occurred in 12 patients (50.0%), most commonly due to transient toxicity or patient preference (Table 2).

After discontinuation of maintenance therapy, post-maintenance treatments were summarized descriptively. Overall, 16 patients (66.7%) received subsequent systemic therapy, most commonly gemcitabine/docetaxel in 13 patients, followed by paclitaxel in two patients and pazopanib in one, while one patient underwent active surveillance.

### 3.3. Efficacy Outcomes

**Figure 1:**
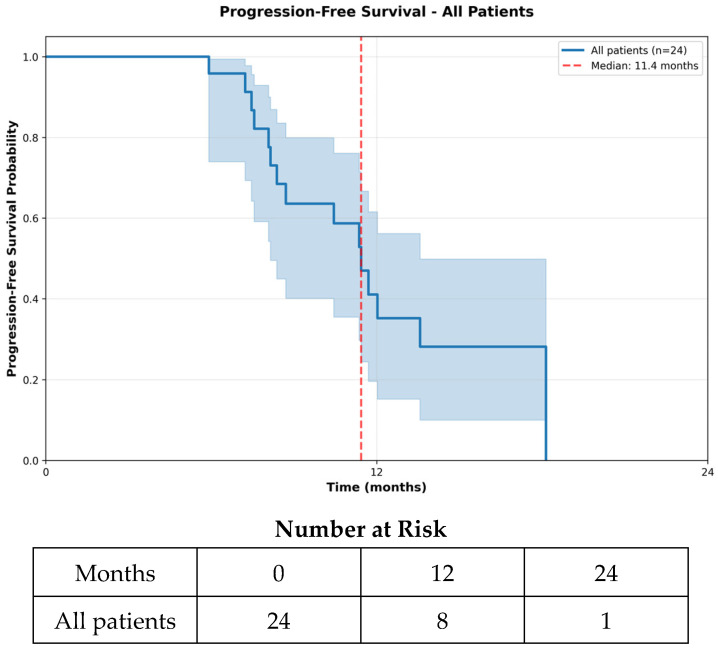
Progression-Free Survival (PFS)—All Patients. Progression-free survival for all patients treated with liposomal doxorubicin is shown, demonstrating the time from treatment initiation to disease progression or death. The Kaplan–Meier curve illustrates the probability of remaining progression-free over time, and the number-at-risk indicates the number of patients under observation at each 12-month interval.

**Figure 2:**
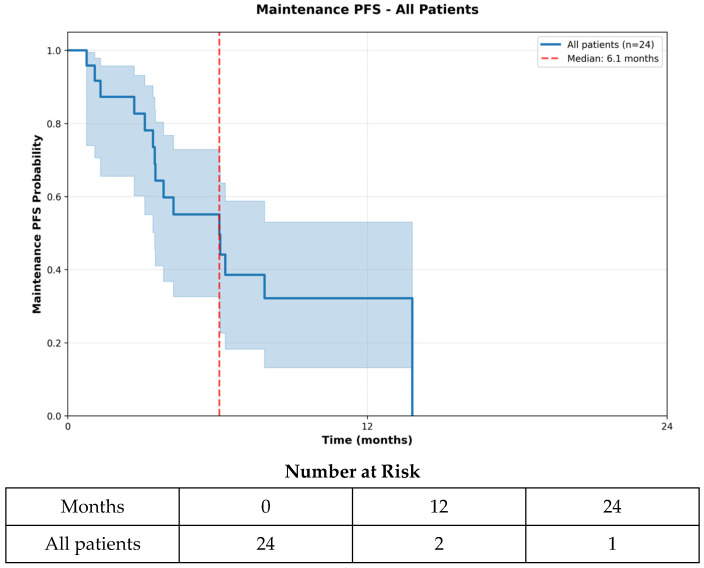
Maintenance PFS—All Patients. Maintenance progression-free survival during liposomal doxorubicin treatment is shown, reflecting disease control during the maintenance phase of therapy. The Kaplan–Meier curve shows the probability of remaining progression-free during maintenance treatment, and the number-at-risk shows patient follow-up at 12-month intervals.

**Figure 3:**
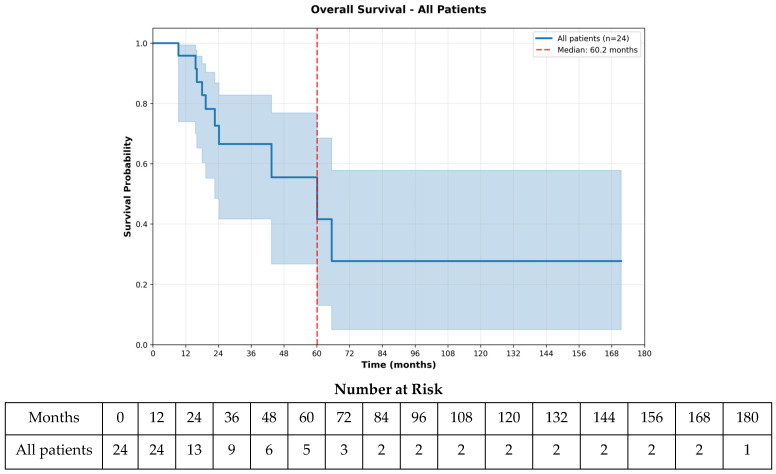
Overall Survival—All Patients. Overall survival for all patients treated with liposomal doxorubicin is displayed, with the Kaplan–Meier curve showing the probability of survival over time. The number-at-risk table shows the number of patients remaining under observation at each 12-month time point, accounting for both events.

The median PFS was 11.4 months (348 days; 95% CI 10.4–14.8 months), while the median mPFS was 6.1 months (185 days; 95% CI 4.2–9.2 months) (Figure 1 and Figure 2). The median OS was 60.2 months (1832 days; 95% CI 24.2–65.6 months) (Figure 3). At the 6-month landmark, 100% of the patients remained alive (Figure S3). Oligoprogression, defined as up to five progressive extracranial metastases under systemic therapy [25], occurred in five patients (20.8%) and was managed with local therapy, including radiation or ablative approaches. Treatment discontinuation occurred in 15 patients (62.5%) due to disease progression, in two patients (8.3%) because of adverse events, in one patient (4.2%) following palliative surgery, and in one patient (4.2%) due to death. At data cutoff, five patients (20.8%) were still receiving therapy. A sensitivity analysis excluding the two patients with best induction response of progressive disease yielded consistent results, with unchanged median OS (60.2 months), PFS (11.4 months), and mPFS (6.1 months).

As a complementary exploratory analysis, OS was also calculated from the start of maintenance liposomal doxorubicin. The median OS from maintenance initiation was 14.2 months (431 days) (Figure S4).

### 3.4. Safety

Commonly reported adverse events were infusion reactions (37.5%), anemia (20.8%), cardiac toxicity (20.8%), fatigue (16.7%), and neutropenia (16.7%). Discontinuation due to adverse events was rare (Table 3).

Cardiac toxicity was assessed descriptively because standardized longitudinal monitoring data were not consistently available. Using a grouped definition (reduced ejection fraction, atrial fibrillation/flutter, and palpitations), 5 of 24 patients (20.8%) experienced a cardiac toxicity event at any point during treatment, including 4 patients (16.7%) during maintenance. The median cumulative induction doxorubicin exposure was 401.3 mg/m^2^ among patients with cardiac toxicity versus 435.0 mg/m^2^ among those without, while the median total anthracycline exposure was 601.3 mg/m^2^ versus 730 mg/m^2^, respectively.

### 3.5. Exploratory Analyses

**Figure 4:**
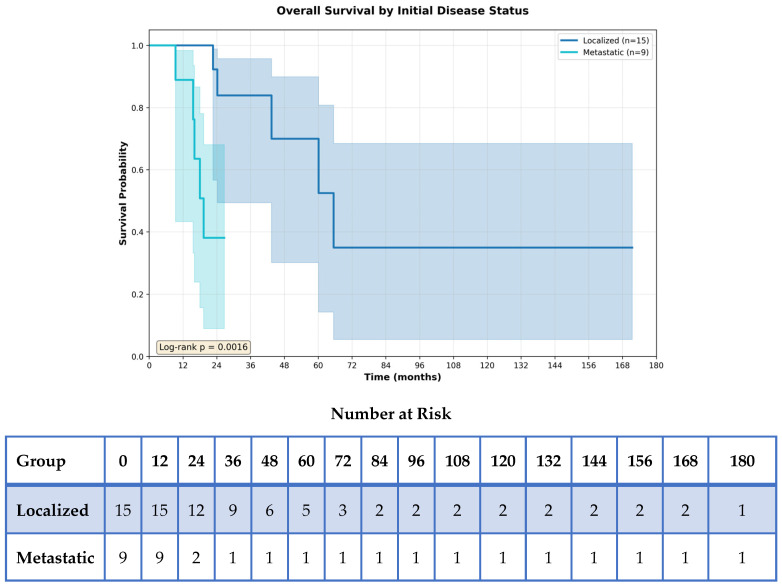
Overall Survival by Initial Disease Status. Overall survival was significantly worse in patients with metastatic disease at initial presentation compared to those with localized disease. The Kaplan–Meier survival curves show a clear separation, with metastatic patients exhibiting substantially reduced survival probability over time. This finding aligns with the Cox regression analysis, which showed initial disease status as the strongest predictor of overall survival. The number-at-risk table shows the distribution of patients at risk over time for each group.

**Figure 5:**
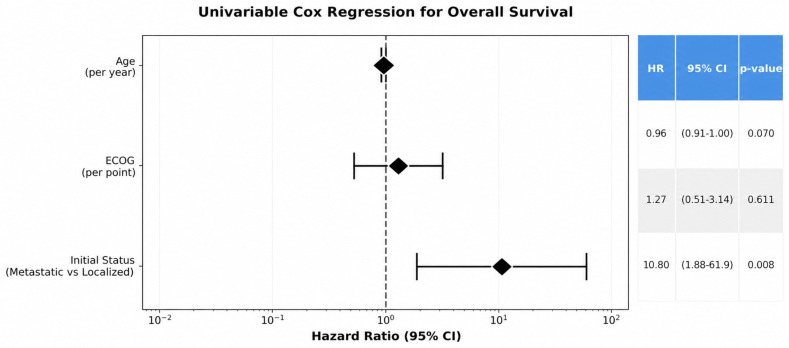
Forest Plot—Univariable Cox Regression for Overall Survival. Forest plot showing results of univariable Cox proportional hazards regression analyses for overall survival. Hazard ratios (HRs) with 95% confidence intervals (CIs) are displayed for age (per year), ECOG performance status (per point), and disease status at diagnosis (metastatic vs. localized). The vertical dashed line represents the null effect (HR = 1.0). *p*-values were derived from Wald tests. Metastatic disease at diagnosis was significantly associated with worse overall survival, whereas age and ECOG performance status were not.

Exploratory analyses were conducted to further characterize factors associated with outcomes. Multivariable Cox proportional hazards models were used to explore factors associated with overall survival. Metastatic disease at diagnosis was significantly associated with worse OS in both univariable (HR 10.8, 95% CI 1.88–61.94, *p* = 0.008) and multivariable models (HR 7.57, 95% CI 1.06–53.92, *p* = 0.043) (Figure 4 and Figure S5). Age (HR = 0.97, *p* = 0.318) and ECOG performance status (HR = 1.04, *p* = 0.943) were not significant in the adjusted model. Additional exploratory analyses showed that ECOG ≥ 2 (HR = 0.46, *p* = 0.463) and sex (HR = 0.79, *p* = 0.715) were not associated with OS (Figure 5).

In addition, exploratory analysis of OS by receipt of post-maintenance systemic therapy (yes vs. no) suggested a difference (log-rank *p* = 0.044); however, these findings should be interpreted cautiously.

Given the marked histologic heterogeneity of the cohort, subtype-specific analyses were not statistically reliable. An exploratory grouped analysis was therefore performed, categorizing patients into leiomyosarcoma, UPS, and other histologies, which did not reveal a clear differential outcome signal for OS, PFS, or mPFS.

## 4. Discussion

To our knowledge, this is the first study specifically examining a maintenance anthracycline approach in advanced STS. First-line doxorubicin-based chemotherapy, alone or in combination, remains the standard of care, but it is usually associated with a significant risk of toxicity, which may limit treatment duration and long-term disease control [7]. The median number of induction cycles in our cohort was six, reflecting current clinical practice that limits cumulative anthracycline exposure [11]. This study suggests that patients with advanced STS who achieve disease control following doxorubicin induction may find maintenance therapy with liposomal doxorubicin to be a feasible and well-tolerated strategy that can sustain clinical benefit.

Compared with historical benchmarks for first-line doxorubicin therapy, where the median PFS is typically 4 to 8 months and the median OS is 12 to 20 months, our study demonstrated a median PFS of 11.4 months and a median OS of 60.2 months, suggesting favorable outcomes in this selected population [7,8,26]. However, our study focused on patients who achieved favorable responses, thereby selecting a population with anthracycline-sensitive disease, whereas pivotal trials establishing doxorubicin as standard therapy included unselected patient populations, which may have affected outcome consistency. As such, the favorable outcomes observed in our cohort may partially reflect this underlying sensitivity rather than the effect of maintenance treatment alone. Although exploratory analyses suggested differences in OS by receipt of post-maintenance therapy, these findings are subject to time-dependent bias and should be interpreted as hypothesis-generating. Furthermore, responder-specific analyses are poorly described in the existing literature, as most studies report outcomes in the intention-to-treat population.

By focusing on patients who benefited from upfront doxorubicin, we were able to better explore whether continued treatment could help preserve that benefit. In this context, the pharmacological characteristics of liposomal doxorubicin, such as decreased hematologic and cardiac toxicity, likely contribute to its tolerability and suitability for extended administration [27].

Furthermore, prior phase II studies have demonstrated clinical activity of liposomal doxorubicin in advanced STS, with manageable toxicities, supporting its role as a reasonable therapeutic option in this setting [28,29]. Efforts to optimize the delivery of anthracyclines in STS have included the development of new agents such as aldoxorubicin and legubicin. In a phase III trial in previously treated advanced STS, aldoxorubicin improved PFS compared with investigator’s choice chemotherapy (median PFS 4.1 vs. 3 months), but it did not ultimately replace traditional doxorubicin as a standard of care [30].

Notably, approximately a quarter of patients experienced oligoprogression that was amenable to local therapy, after which they were able to continue with the maintenance therapy. This suggests that liposomal doxorubicin may maintain disease control even beyond locally treated oligoprogression. This finding is consistent with the study by Willmann et al., which compared continuation of the same systemic therapy versus switching to a new regimen after stereotactic body radiotherapy (SBRT) for oligoprogressive disease across several tumor types and showed comparable oncological outcomes [25]. Taken together, these may support the rationale and safety of maintaining the same systemic therapy post local treatment in patients with oligoprogression, highlighting a subset of patients with potential for durable benefit.

New data on alternative maintenance strategies further support the potential of long-term post-induction treatment for advanced STS. In patients with unresectable or metastatic STS, a recent phase II trial by Li et al. assessed epirubicin plus anlotinib followed by maintenance anlotinib. The results showed a disease control rate of 80% and a median PFS of 11.5 months. With few discontinuations related to intolerance and most adverse events being grade 1 or 2, toxicity was manageable [31]. Similarly, the EREMISS trial evaluated maintenance regorafenib following initial chemotherapy in advanced STS, additionally supporting the feasibility of tyrosine kinase inhibitor (TKI)-based maintenance strategies in this setting [32]. In addition, the T-DIS randomized phase II trial compared ongoing use of trabectedin versus interruption after six cycles and concluded that continuing trabectedin until progression resulted in a significantly higher 6-month PFS rate (51.9% vs. 23.1%) with acceptable safety [33]. In the leiomyosarcoma subgroup, the LMS-04 trial demonstrated that induction chemotherapy with doxorubicin plus trabectedin, followed by trabectedin maintenance, yielded longer PFS and OS than doxorubicin alone [34]. These results are consistent with those in our cohort, indicating that maintenance approaches, whether using multitargeted TKI- or chemotherapy-based regimens, may be a valuable means of prolonging disease stability after first-line therapy. Although maintenance strategies are commonly used in clinical practice, no standard approach has been established for advanced STS, and direct comparisons with maintenance liposomal doxorubicin are not possible due to a lack of comparative data.

However, it is essential to mention the substantial financial burden associated with TKI-based maintenance strategies, as these drugs can be costly and limit real-world feasibility [35]. In contrast, anthracycline-based approaches are generally more affordable and widely accessible, rendering them a more practical option in many healthcare settings. Moreover, our study showed a low discontinuation rate due to adverse events, suggesting a generally favorable tolerability profile for prolonged liposomal doxorubicin use.

Although limited by its retrospective nature, small sample size, and histologic heterogeneity, this analysis adds to the scarce evidence exploring maintenance chemotherapy in STS. The absence of a comparator group limits the ability to draw causal inferences and introduces potential selection bias. Cardiac toxicity was observed in a minority of patients, without an apparent association with cumulative anthracycline exposure; however, these findings are limited by the absence of standardized cardiac monitoring. Remarkably, maintenance strategies in STS remain poorly defined, and most systemic options beyond first-line therapy offer modest benefit with cumulative toxicity [36,37]. In this context, our findings support the concept that prolonged disease control may be achievable with regimens that balance efficacy with long-term tolerability.

Furthermore, the favorable toxicity profile of liposomal doxorubicin aligns with its pharmacological advantages, particularly reduced cardiotoxicity and myelosuppression, making it more suitable for extended administration. The patient’s ability to remain on therapy underscores its role as a maintenance agent.

## 5. Conclusions

In conclusion, the data presented suggest that liposomal doxorubicin as a maintenance therapy in patients with STS who achieve response or disease control after induction with doxorubicin is a feasible and generally well-tolerated therapeutic strategy. This approach may allow for continued disease management with a manageable toxicity profile, making it appealing for patients with limited subsequent treatment options, as is the case with STS. Compared to TKIs, liposomal doxorubicin may offer a more readily available and cost-conscious alternative in routine clinical practice.

Importantly, this study provides real-world evidence of outcomes in routine clinical practice, highlighting the feasibility of implementing anthracycline-based maintenance strategies across a varied patient population. Our study adds to the limited but growing evidence supporting maintenance chemotherapy in STS and underscores the potential for extended disease control, including in patients experiencing oligoprogression. However, given the retrospective design, small sample size, and lack of a comparator arm, these findings are hypothesis-generating and should not be interpreted as evidence of efficacy.

Future prospective studies should focus on detecting biomarkers predictive of sustained response, assessing quality-of-life outcomes, incorporating cardiac safety monitoring, and defining the most appropriate patient selection criteria. These efforts are necessary to create evidence-based guidelines that optimize both efficacy and tolerability in this challenging population.

## Figures and Tables

**Table 1:** Baseline patient and disease characteristics (*n* = 24).

Characteristic	n (%)/Median (Range)
**Age, years**	61 (36–82)
**Sex**	
Male	15 (62.5)
Female	9 (37.5)
**ECOG performance status**	
0–1	19 (79.2)
≥2	5 (20.8)
**Disease stage at diagnosis**	
Localized	15 (62.5)
Metastatic	9 (37.5)
**Histologic subtype**	
Leiomyosarcoma	7 (29.2)
UPS	7 (29.2)
MPNST	2 (8.3)
Solitary fibrous tumor	2 (8.3)
Other subtypes	6 (25)

Data are presented as *n* (%). Age is reported as median (range). ECOG = Eastern Cooperative Oncology Group; UPS = undifferentiated pleomorphic sarcoma; MPNST = malignant peripheral nerve sheath tumor. “Other subtypes” include histologies with one patient each.

**Table 2:** Treatment exposure and management.

Parameter	n (%)/Median (Range)
**Induction therapy regimen**	
Single-agent doxorubicin	18 (75.0)
Doxorubicin combination	6 (25.0)
**Number of induction cycles**	6 (1–12)
**Best response to induction**	
Partial response	9 (37.5)
Stable disease	13 (54.2)
Progressive disease	2 (8.3)
**Maintenance therapy**	
Liposomal doxorubicin 40 mg/m^2^ q28 days	23 (96.0)
**Number of maintenance cycles**	5 (1–19)
**Dose reductions**	2 (8.3)
**Treatment interruptions**	12 (50.0)
**Oligoprogression treated locally**	5 (20.8)
**Discontinuation reason**	
Disease progression	15 (62.5)
Adverse events	2 (8.3)
Other (death, surgery)	2 (8.3)
Ongoing at data cutoff	5 (20.8)

Data are presented as *n* (%). Continuous variables are reported as median (range). q28 days = every 28 days. Oligoprogression refers to limited-site disease progression. “Other” reasons for discontinuation include death and surgery. Data cutoff reflects the date of last follow-up for ongoing patients.

**Table 3:** Treatment-related adverse events.

Adverse Event	n (%)
Infusion reactions	9 (37.5%)
Anemia	5 (20.8%)
Cardiac toxicity	5 (20.8%)
Fatigue	4 (16.7%)
Neutropenia	4 (16.7%)
Skin changes/Rash	2 (8.3%)

Data are presented as *n* (%). Adverse events were assessed during maintenance liposomal doxorubicin therapy and recorded according to routine clinical documentation. Cardiac toxicity includes reduced ejection fraction and clinically significant arrhythmias. Events are reported regardless of grade.

## Data Availability

The datasets used and/or analyzed during the current study are available from the corresponding author upon reasonable request.

## Supplementary Materials

The following supporting information can be downloaded at https://www.mdpi.com/article/10.3390/curroncol33050260/s1, Figure S1: Baseline Patient Characteristics; Figure S2: Distribution of Liposomal Doxorubicin Treatment Cycles; Figure S3: 6-Month Landmark Analysis for Overall Survival; Figure S4: Overall Survival from Start of Maintenance Therapy; Figure S5: Overall Survival by ECOG Performance Status.

## Abbreviations

The following abbreviations are used in this manuscript:

STS	Soft tissue sarcoma
OS	Overall survival
PFS	Progression-free survival
mPFS	Maintenance progression-free survival
