# Supplementary material for: Maintenance Liposomal Doxorubicin Following Induction Doxorubicin in Soft Tissue Sarcoma: A Single-Center Observational Study
Source: Curr Oncol. 2026 Apr 30;33(5):260. doi: 10.3390/curroncol33050260 (PMC13206444; doi:10.3390/curroncol33050260)
Supplement: Supplementary file 1 [file curroncol-33-00260-s001.zip › curroncol-4248066-supplementary.pdf]

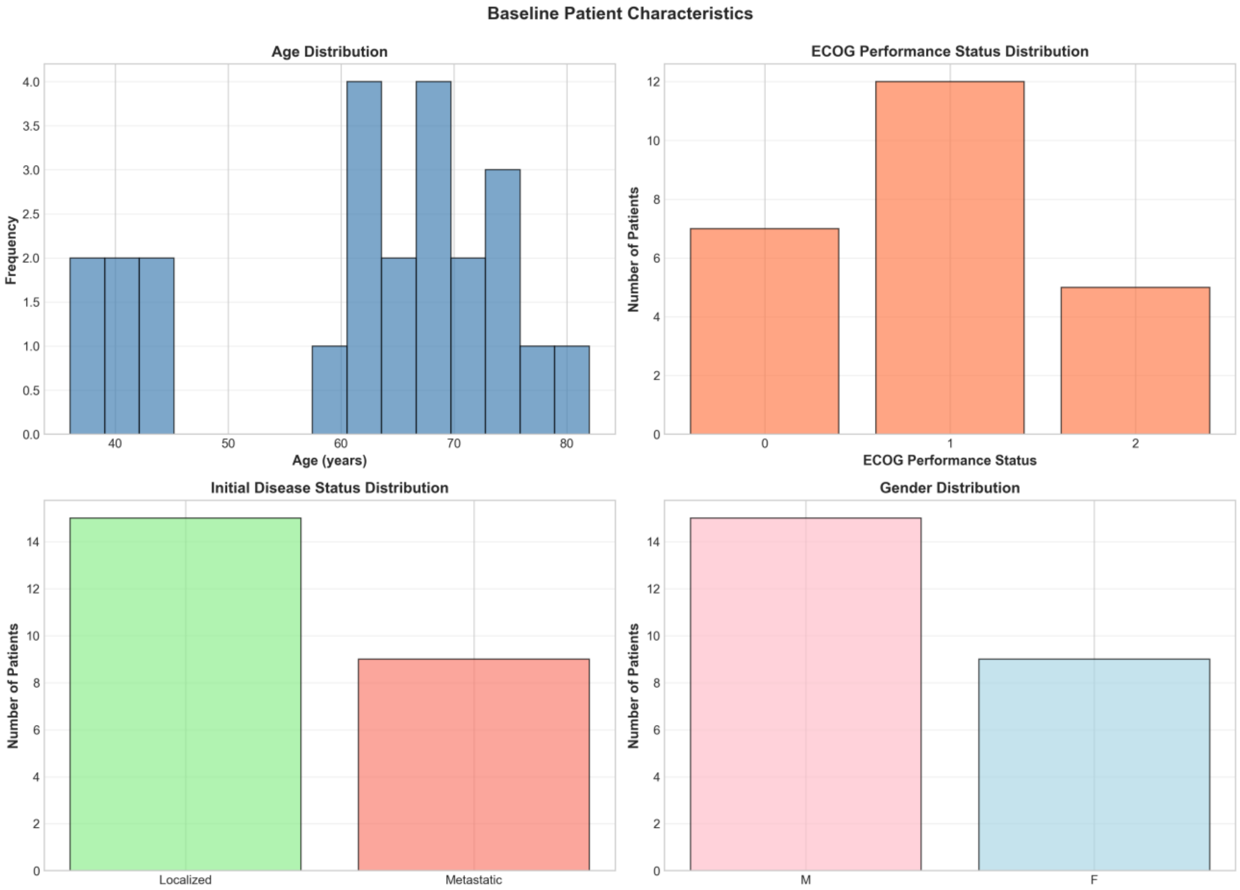

Figure S1. Baseline Patient Characteristics. Baseline characteristics demonstrate the demographic and clinical profile of the study population. The distributions show the age range, performance status, disease stage, and gender representation among patients receiving liposomal doxorubicin treatment.

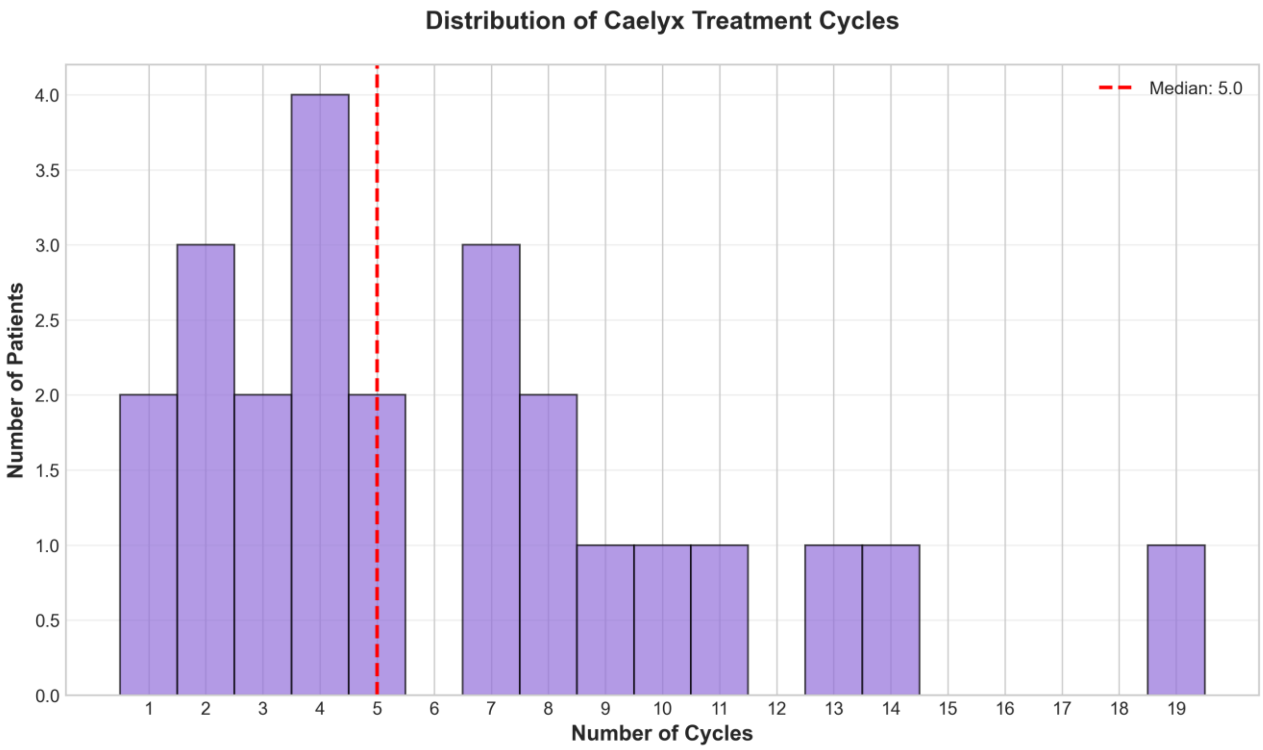

Figure S2. Distribution of Liposomal Doxorubicin Treatment Cycles. The histogram shows the distribution of completed treatment cycles, with a median of 5.0 cycles (mean: 6.4). This distribution reflects treatment tolerability and duration of therapy, with variability potentially influenced by treatment response, adverse events, or disease progression.

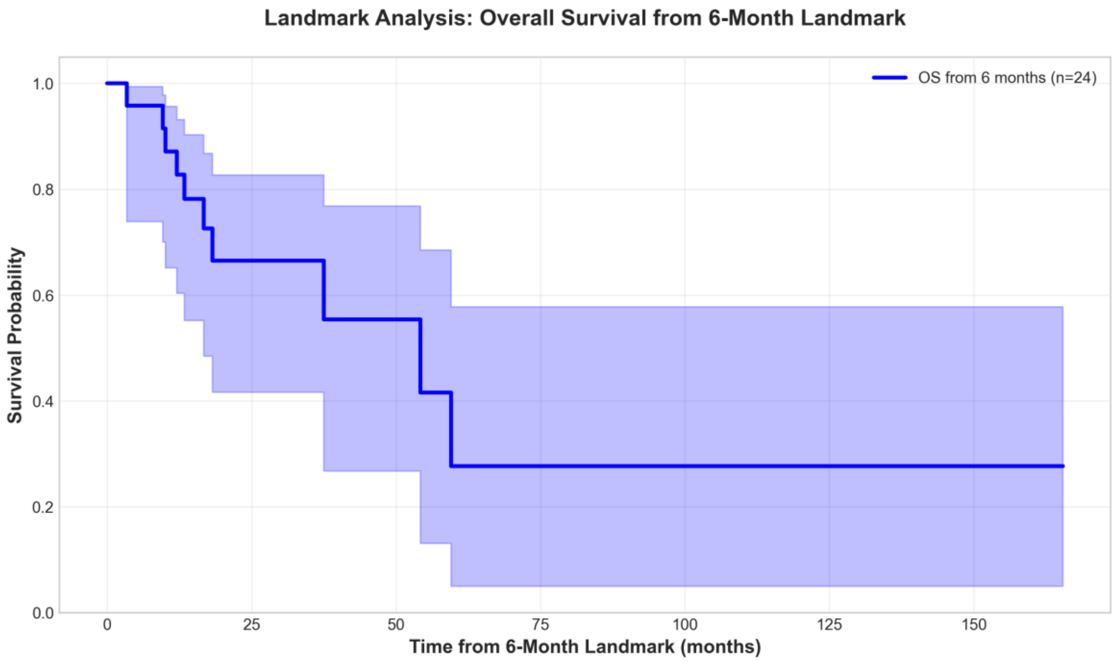

Figure S3. 6-Month Landmark Analysis for Overall Survival. Landmark analysis at 6 months demonstrates survival outcomes for patients who remained alive at this timepoint, providing insight into longer-term prognosis. Of 24 evaluable patients, 24 (100.0%) were alive at the 6-month landmark. This analysis helps address potential lead-time bias and provides prognostic information for patients who achieve initial disease control.

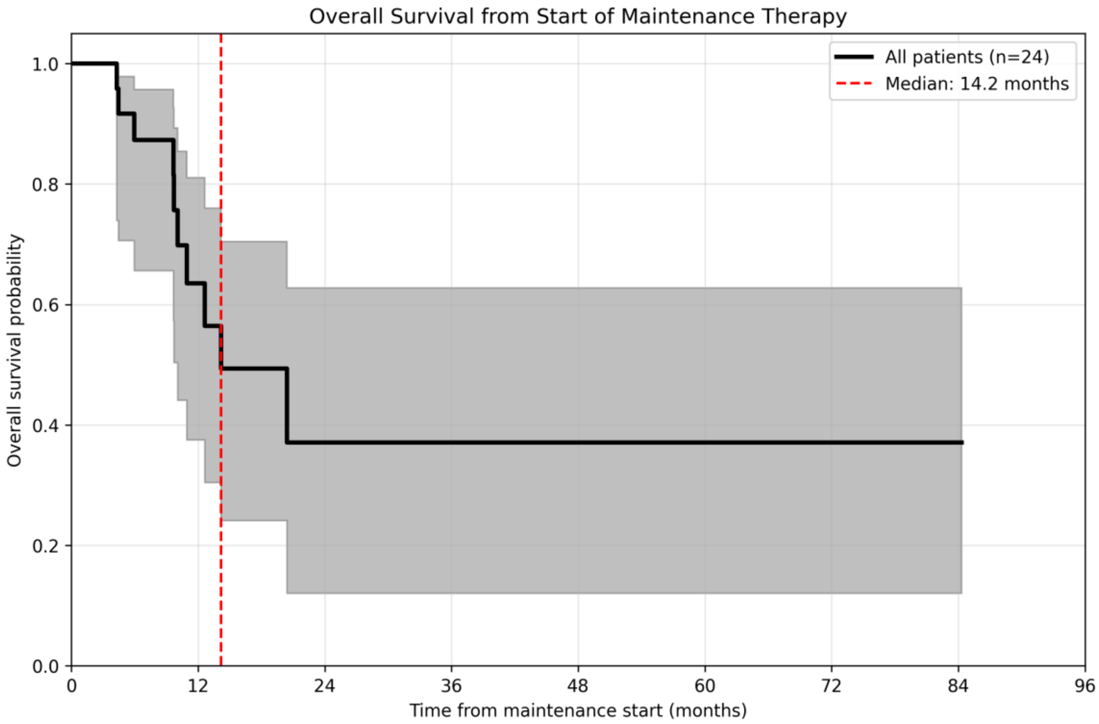

Figure S4. Overall Survival from Start of Maintenance Therapy. Exploratory supplementary figure: Overall survival calculated from the start of maintenance therapy.

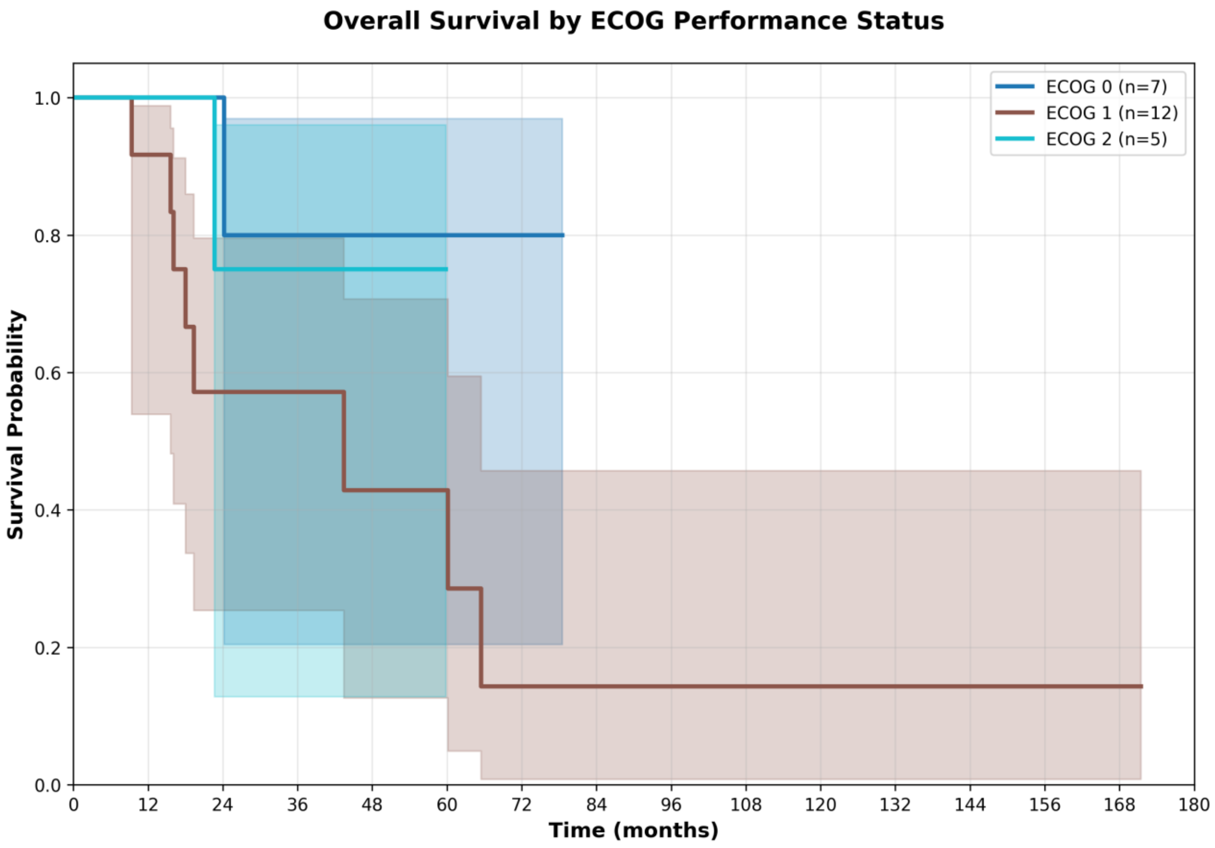

Number at Risk

Group	0	12	24	36	48	60	72	84	96	108	120	132	144	156	168	180
**ECOG 0**	7	7	6	4	2	2	2	1	1	1	1	1	1	1	1	1
**ECOG 1**	12	12	5	5	4	4	2	2	2	2	2	2	2	2	2	1
**ECOG 2**	5	5	4	2	2	1	1	1	1	1	1	1	1	1	1	1

Figure S5. OS by ECOG Performance Status. Patients with higher ECOG performance status scores showed a trend toward decreased overall survival, although this did not reach statistical significance. The survival curves suggest that baseline functional status may influence outcomes, with better performance status associated with improved survival. The number-at-risk display patient distribution over time by ECOG group.
